# The Presence of White Matter Lesions Is Associated With the Fibrosis Severity of Nonalcoholic Fatty Liver Disease

**DOI:** 10.1097/MD.0000000000003446

**Published:** 2016-04-22

**Authors:** Salvatore Petta, Antonino Tuttolomondo, Cesare Gagliardo, Rita Zafonte, Giuseppe Brancatelli, Daniela Cabibi, Calogero Cammà, Vito Di Marco, Luigi Galvano, Giuseppe La Tona, Anna Licata, Franco Magliozzo, Carlo Maida, Giulio Marchesini, Giovanni Merlino, Massimo Midiri, Gaspare Parrinello, Daniele Torres, Antonio Pinto, Antonio Craxì

**Affiliations:** From the Sezione di Gastroenterologia e Epatologia (SP, CC, VDM, AL, AC), Sezione di Medicina Interna, DiBiMIS, University of Palermo (AT, CM, GP, DT, AP), Sezione di Scienze Radiologiche, Dipartimento di Biopatologia e Biotecnologie Mediche (DI.BI.MED.), Università degli Studi di Palermo (CG, GB, GLT, MM), Medicina Generale Palermo (RZ, LG, FM, GM), Cattedra di Anatomia Patologica, University of Palermo, Palermo (DC), and Dipartimento di Scienze Mediche e Chirurgiche, “Alma Mater Studiorum,” Università di Bologna, Bologna, Italy (GM).

## Abstract

We tested whether nonalcoholic fatty liver disease (NAFLD) and/or its histological severity are associated with vascular white matter lesions (WML) in patients with biopsy-proven NAFLD and in non-NAFLD controls.

Data were recorded in 79 consecutive biopsy-proven NAFLD, and in 82 controls with normal ALT and no history of chronic liver diseases, without ultrasonographic evidence of steatosis and liver stiffness value <6 KPa. All subjects underwent magnetic resonance assessment and WML were classified according to the Fazekas score as absent (0/III), or present (mild I/III; moderate II/III, and severe I/III). For the purpose of analyses, all controls were considered without NASH and without F2–F4 liver fibrosis.

WML were found in 26.7% of the entire cohort (43/161), of moderate–severe grade in only 6 cases. The prevalence was similar in NAFLD versus no-NAFLD (29.1% vs 24.3%; *P* = 0.49), but higher in NASH vs no-NASH (37.7% vs 21.2%, *P* = 0.02) and F2–F4 vs F0-F1 fibrosis (47.3% vs 20.3%, *P* = 0.001). In both the entire cohort and in NAFLD, only female gender (OR 4.37, 95% CI: 1.79–10.6, *P* = 0.001; and OR 5.21, 95% CI: 1.39–19.6, *P* = 0.01), age > 45 years (OR 3.09, 95% CI: 1.06–9.06, *P* = 0.03; and OR 11.1, 95% CI: 1.14–108.7, *P* = 0.03), and F2–F4 fibrosis (OR 3.36, 95% CI: 1.29–8.73, *P* = 0.01; and OR 5.34, 95% CI: 1.40–20.3, *P* = 0.01) were independently associated with WML (mostly of mild grade) by multivariate analysis. Among NAFLD, the prevalence of WML progressively increased from patients without (1/18; 5.5%), or with 1 (1/17, 5.8%), to those with 2 (9/30; 30%) and further to those with 3 (12/14; 85.7%) risk factors.

The presence of WML is not associated with NAFLD, but with metabolic diseases in general, and fibrosis severity of NAFLD. Clinical implications of this issue need to be assessed by longitudinal studies.

## INTRODUCTION

The prevalence of nonalcoholic fatty liver disease (NAFLD) is increasing worldwide in parallel to the growing pandemic of overweight and obesity.^1^ This picture accounts for NAFLD as the most common cause of chronic liver disease,^2^ as a dramatically growing risk factor of hepatocellular carcinoma,^3^ and as an indication for liver transplantation.^4^ Long-term prospective studies looking at the natural history of NAFLD showed that, although patients with fatty liver accumulation are at high risk of liver-related mortality, cardiovascular events remain the most common cause of death.^5,6^ Accordingly, a growing body of literature reported a higher risk of early asymptomatic cardiovascular alterations and/or overt cardiovascular disease in NAFLD. Specifically, NAFLD, diagnosed either by ultrasonography or by liver biopsy, has been associated with a higher prevalence of low coronary flow reserve,^7^ coronary calcification,^8^ and carotid atherosclerosis,^9–11^ well-before the occurrence of cardiovascular events. These alterations have been partly associated with the severity of liver damage, measured by both lobular inflammation and fibrosis. Along this line, both cross-sectional and prospective studies showed an association between NAFLD and the presence/extent of coronary and peripheral cardiovascular events,^12^ after adjustment for cardiometabolic confounders.^13^ Available data suggested that NAFLD patients are also at increased risk for the occurrence of cerebrovascular events.^12,13^

Cerebral white matter lesions (WML) are considered manifestations of cerebral small vessel disease,^14,15^ leading to an increased risk of stroke, cognitive decline, dementia, disability, and mortality.^16,17^ The prevalence of WML ranges from 11% to 21% in adults aged around 64% to 94% at age over 80,^18^ and it is highly associated with the distribution of the main cardiovascular risk factors, namely older age, hypertension, diabetes, and dyslipidemia,^19–23^ as well as with the grade of hepatic encephalopathy in cirrhotic patients.^24^

Considering the link between NAFLD and both cerebrovascular events and metabolic alterations, we aimed to assess whether NAFLD and/or its histological severity are associated with the presence of WML in a mixed cohort of patients with biopsy-proven NAFLD and of controls without fatty liver.

## PATIENTS AND METHODS

### Patients

The study involved 79 consecutive patients with biopsy-proven NAFLD and 82 individuals without fatty liver.

NAFLD patients were recruited at the Gastrointestinal & Liver Unit of Palermo University Hospital, and fulfilled all the inclusion and exclusion criteria detailed below. Inclusion criterion was a histological diagnosis of NAFLD on a liver biopsy done less than 6 months before enrollment, showing steatosis (>5% of hepatocytes) with or without necroinflammation and/or fibrosis including cirrhosis. Exclusion criteria were decompensated cirrhosis (jaundice, presence of ascites, or encephalopathy); hepatocellular carcinoma; liver disease of different or mixed etiology; human immunodeficiency virus infection; history of heart diseases (TIA, stroke, angina, myocardial infarction, right or left hearth decompensation); and active intravenous drug addiction.

Control individuals were referred from general practitioners. They were part of an ongoing project aimed at assessing cardiovascular risk and liver damage in the general population, according to the presence of NAFLD at ultrasounds. Among 209 tested individuals, 120 had no steatosis but fulfilled all the criteria indicated above; 82 of them accepted to be submitted to cerebral MR. They had no previous history of symptomatic cardiovascular disease (transient ischemic attack, stroke, angina, myocardial infarction, right or left ventricular dysfunction), no history of chronic liver disease, no evidence of viral infection (anti-HCV, anti-HIV, and HBsAg negativity), alcohol consumption <20 g/day during the previous year (evaluated by a specific questionnaire), normal ALT values (<37 UI/L), no ultrasonographic (US) evidence of steatosis, and a liver stiffness value <6 KPa. Biochemical analyses were performed in the same central laboratory used for NAFLD patients, and history data were obtained using a standardized interview in both cases and controls.

The study was carried out in accordance with the principles of the Helsinki Declaration and its appendices, and with local and national laws. Approval was obtained from the AOUP Paolo Giaccone in Palermo and its Ethics Committee, and written informed consent was obtained from all patients.

### Clinical and Laboratory Assessment

Clinical and anthropometric data were collected at the time of enrollment. The diagnosis of arterial hypertension^25^ and type 2 diabetes^26^ were based on standard criteria. Menopause was defined as no menstrual periods for 12 consecutive months.

A 12-hour overnight fasting blood sample was drawn at the time of enrollment to determine the serum levels of ALT, total cholesterol, HDL-cholesterol, triglycerides, plasma glucose, insulin (for NAFLD only), and platelet count (for NAFLD only). Insulin resistance (IR) was determined according to the homeostasis model assessment (HOMA) method.^27^

### Ultrasound Assessment

Ultrasound assessment was performed in fasting subjects, on the day of liver biopsy for NAFLD, and on the day of enrollment for the controls, by one operator trained for ultrasound techniques and particularly dedicated to liver examination. A real-time Hitachi H21 apparatus with a 2 to 5 MHz, convex, multifrequency probe was used. Presence of hepatic steatosis was defined by detection of Bright Liver Echo pattern (BLEP), that is, fine, packed, and high amplitude echoes, with consequent brightness of liver, increase in liver–kidney contrast and possible evidence of vascular blurring and deep attenuation signs.^28^

### Liver Stiffness Measurement (LSM)

Transient elastography was performed with the FibroScan (Echosens, Paris, France) medical device, using the M probe (also named as standard probe), following the ultrasound examination, by trained operators who had previously performed at least 300 determinations in patients with chronic liver disease.^29^

### Carotid Artery Evaluation

Carotid atherosclerosis was evaluated by an expert physician (D.T.) blinded as to the characteristic of patients, using a high-resolution B-mode ultrasonography equipped with a multifrequency linear probe.

Carotid arteries were investigated as previous described.^11^ A carotid plaque was defined as a focal thickening >1.3 mm at the level of either common and internal carotid arteries or bifurcations.^11^

### White Matter Lesions

All subjects underwent brain scan using a 1.5 T MRI scanner (Signa HDxt; GE Medical System, Milwaukee, WI) with a dedicated phased-array head coil (8ch Brain HD). The protocol was as follows: sagittal 3D T2w FLuid Attenuated Inversion Recover with fat-saturation (FLAIR fat-sat; spatial resolution 1.0 mm × 1.0 mm × 1.2 mm; acquisition matrix 224 × 224; TR 6000ms; ET 125.621ms; IT 1864ms; FA 90; ETL 130; NEX 1), sagittal 3D T1w Fast Spoiled Gradient Echo (FSPGR; spatial resolution 1.1 mm × 1.1 mm × 0.6 mm; acquisition matrix 256 × 256; TR 12.368ms; ET 5.088ms; IT 450ms; FA 90; ETL 1; NEX 1), axial 2D T2w Fast Recovery Fast Spin Echo (FRFSE; acquisition matrix 384 × 224; slice thickness 3 mm; TR 3740ms; TE 103.4ms; ETL 15; NEX 4), axial 3D T2∗-based multiecho gradient-recalled echo (Susceptibility-Weighted ANgiography, SWAN; slice thickness 3 mm, spacing 1.5 mm, matrix 288 × 224; TR 78.5ms; ET 47.636ms; FA 15; ETL 10; NEX 0.69454) and axial Echo-Planar Diffusion Weighted Imaging (EP-DWI; acquisition matrix 128 × 128; slice thickness 3 mm; TR 7000ms; TE 98ms; NEX 2; using a b value of 0 and 1000 s/mm^2^). Axial and coronal 3 mm thick Multi-Planar Reconstructions (MPR) where obtained from the 3D T2w FLAIR fat-sat and from the 3D T1w FSPGR sequences using a mean algorithm. Axial 3 mm thick MPR where obtained from the 3D T2∗ SWAN sequence using a minimum Intensity Projection (minIP) algorithm. The sagittal plane was parallel to the intercommissure line (ICL), the axial plane was parallel to the anterior commissure–posterior commissure (AC–PC) line and the coronal plane was perpendicular to the previous one.

White matter lesions (WML) were defined as areas (≥5 mm in diameter) with high signal intensities on T2-image. Lacunae were defined as spheroid areas of tissue loss, fluid-filled, of ≥3 and ≤15 mm in diameter, with high signal on T2w and low signal on FLAIR images, iso-hypointense signal on T1-weighted images, and mostly with an hyperintense rim around the cavity on FLAIR images (this hyperintense rim helps differentiating lacunae from dilated Virchow-Robin spaces which have no hyperintense rim on FLAIR images). Cerebral microbleeds have been identified as multiple round foci of marked loss of signal intensity on SWAN sequences. The absence of diffusion restriction on Apparent Diffusion Coefficient maps from the EP-DWI sequence excluded acute ischemic strokes together with characteristic neuroradiological findings on other pulse sequences.

Since the aim of this work was to investigate possible correlations between cerebrovascular lesions (regardless of their location) and NAFLD, a simplified Fazekas visual rating scale for WML was used. Cerebrovascular-related lesions were graded as follows: 0/III = absence of cerebrovascular-related findings, 1/III = mild findings (punctate WML foci and/or periventricular “caps” or pencil-thin lining), 2/III = moderate findings (beginning confluence of WML foci and/or smooth periventricular “halo”), 3/III = severe findings (large confluent WML and/or irregular periventricular areas extending into the deep white matter). All MRI were done by an expert single operator (C.G.) blinded of characteristics of cases and controls.

### Assessment of Cognitive Function (Mini-Mental State Examination)

The Mini-Mental State Examination (MMSE)—a tool that can be used to systematically and thoroughly assess mental status—was administered. It is an 11-question measure that tests 5 areas of cognitive function (orientation, registration, attention and calculation, recall, and language). Maximum score is 30 whereas a MMSE value <24 (23 or lower) is indicative of cognitive impairment.^30^

### Assessment of Histology

Slides of NAFLD were coded and read by 1 pathologist (D.C.), who was unaware of the patients’ identity and history. A minimum length of 15 mm of biopsy specimen or the presence of at least 10 complete portal tracts was required.^31^ Steatosis was assessed as the percentage of hepatocytes containing fat droplets (minimum 5%). The Kleiner classification^32^ was used to grade steatosis, lobular inflammation, and hepatocellular ballooning, and to stage fibrosis from 0 to 4. NASH was considered to be present when steatosis, ballooning, and lobular inflammation were all present.

### Statistics

The study had the power to include 8 potential predictors in the multivariate model for WML in a cohort of patients with and without NAFLD with at least 40 patients with WML, that is, the expected 25% of the entire cohort.

Continuous variables were summarized as mean ± standard deviation and categorical variables as percentage. For purpose of the analysis, rationally, patients without steatosis were considered without both NASH and F2–F4 fibrosis as well. A multiple logistic regression model was used to assess the factors independently associated with presence of WML in the entire cohort and in NAFLD patients only. As candidate risk factors, we selected age, gender, BMI, baseline ALT, LSM, triglycerides, total and HDL cholesterol, blood glucose, diabetes, arterial hypertension, IMT, carotid plaques, smoking, NAFLD, NASH, and F2–F4 fibrosis. Insulin and HOMA were added in the NAFLD model.

Variables associated with the dependent variable at univariate analysis (probability threshold, *P* ≤ 0.10) were included in the multivariate regression models. Regression analyses were performed using SPSS.

## RESULTS

Comparison of patients with NAFLD and individuals without steatosis is reported in Table 1.

**TABLE 1:**
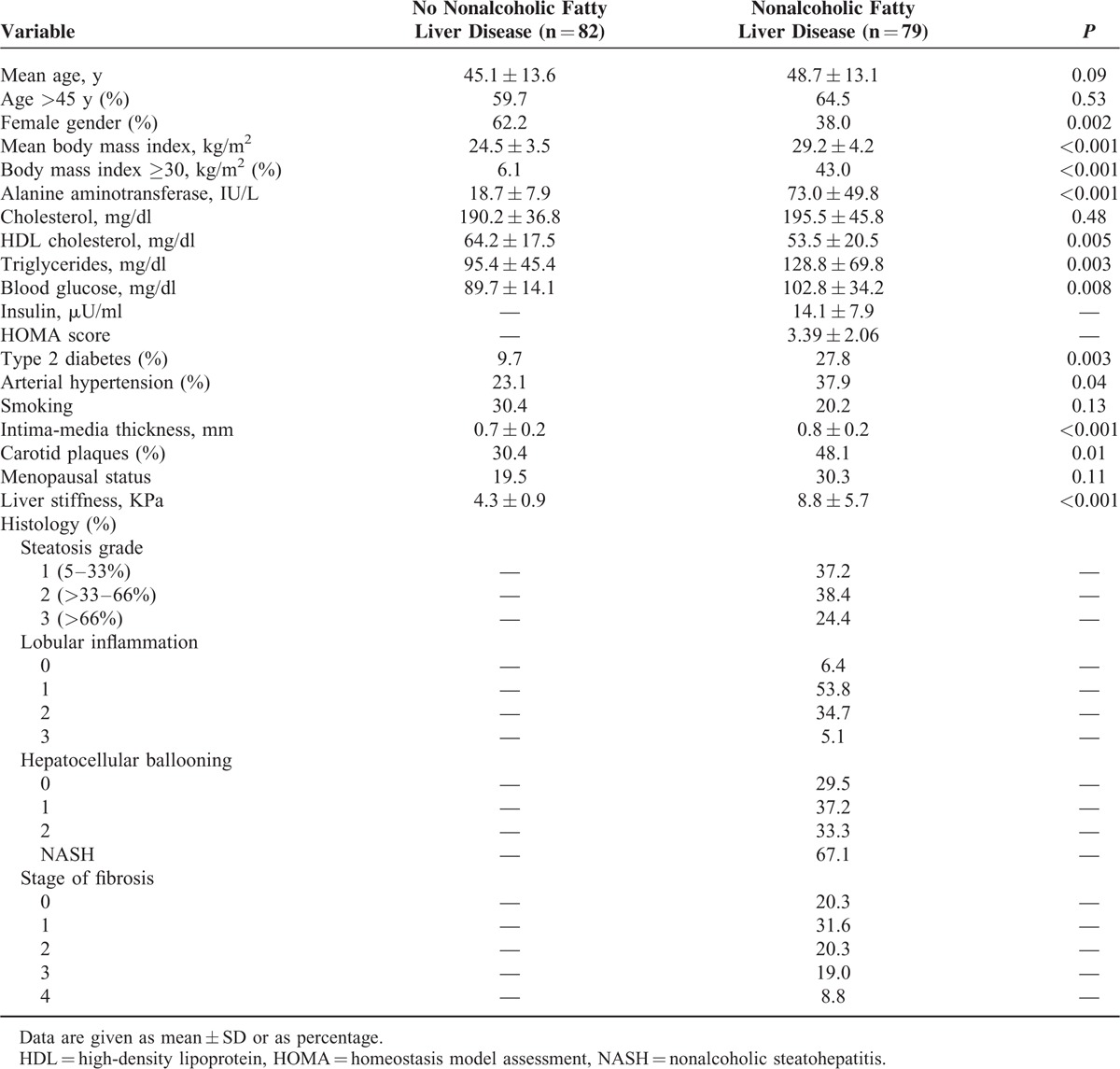
Demographic, Laboratory, Metabolic, and Histological Features of 79 Consecutive Patients With Nonalcoholic Fatty Liver Disease and 82 Individuals Without Steatosis

All individual without steatosis had normal ALT levels, and liver stiffness values less than 6 KPa. Among NAFLD patients, NASH was diagnosed in 67% of cases, and half of patients had F2–F4 fibrosis.

### White Matter Lesions and NAFLD

**FIGURE 1:**
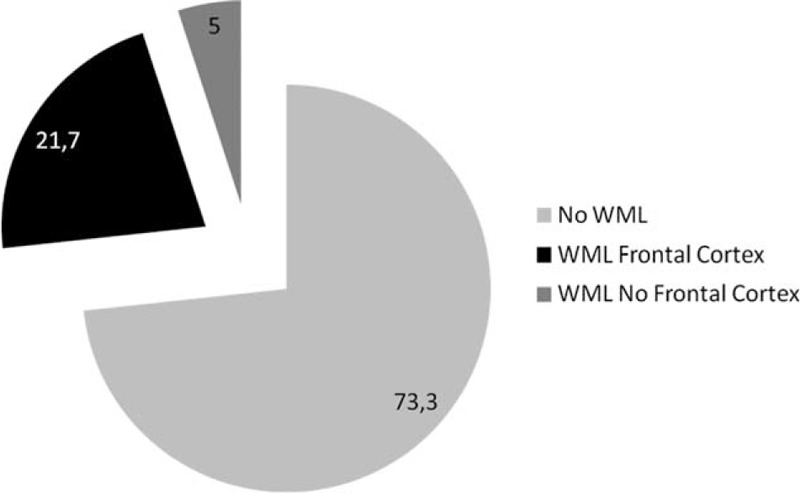
Prevalence and topographic distribution of white matter lesions in the entire cohort of patients with fatty liver and controls without steatosis.

WML were found in 26.7% of the entire cohort (43/161), of moderate–severe grade in only 6 patients. Notably, among patients with WML, the greater proportion of them (81.3%) had lesions in the frontal cortex (Figure 1); the 13 patients with both diabetes and WML had ischemic vascular lesions in the frontal region.

The presence of WML was significantly associated with older age, female gender, type 2 diabetes, arterial hypertension, and carotid plaques (*P* < 0.10), not with the presence of NAFLD (29.1% vs 24.3%; *P* = 0.49). Notably, assuming that patients without steatosis had neither NASH nor F2–F4 fibrosis, we found that the presence of WML was significantly higher in patients with NASH (37.7% vs 21.2%; *P* = 0.02), and in those with F2–F4 fibrosis (47.3% vs 20.3%; *P* = 0.001) when compared with controls. At multivariate logistic regression analysis, WML were significantly associated with age ≥45 (OR 3.09, 95% CI: 1.06–9.06; *P* = 0.03), female gender (OR 4.37, 95% CI: 1.79–10.6; *P* = 0.001), and presence of F2–F4 fibrosis (OR 3.36, 95% CI: 1.29–8.73; *P* = 0.01) (Table 2). When considering age as continuous instead of categorical variable we confirmed the independent link between F2–F4 fibrosis and WML (OR 3.24, 95% CI: 1.23–8.54; *P* = 0.01). When NASH was added as independent variable in the model, we confirmed F2–F4 fibrosis (OR 4.46, 95% CI: 1.04–20.6; *P* = 0.04), but not NASH (OR 0.67, 95% CI: 0.17–2.65; *P* = 0.57) as independent predictor of WML. When the variable “NAFLD” was forced into the model replacing F2–F4 fibrosis, we confirmed the lack of association between NAFLD and WML (OR 1.29, 95% CI: 0.55–3.00; *P* = 0.54).

**TABLE 2:**
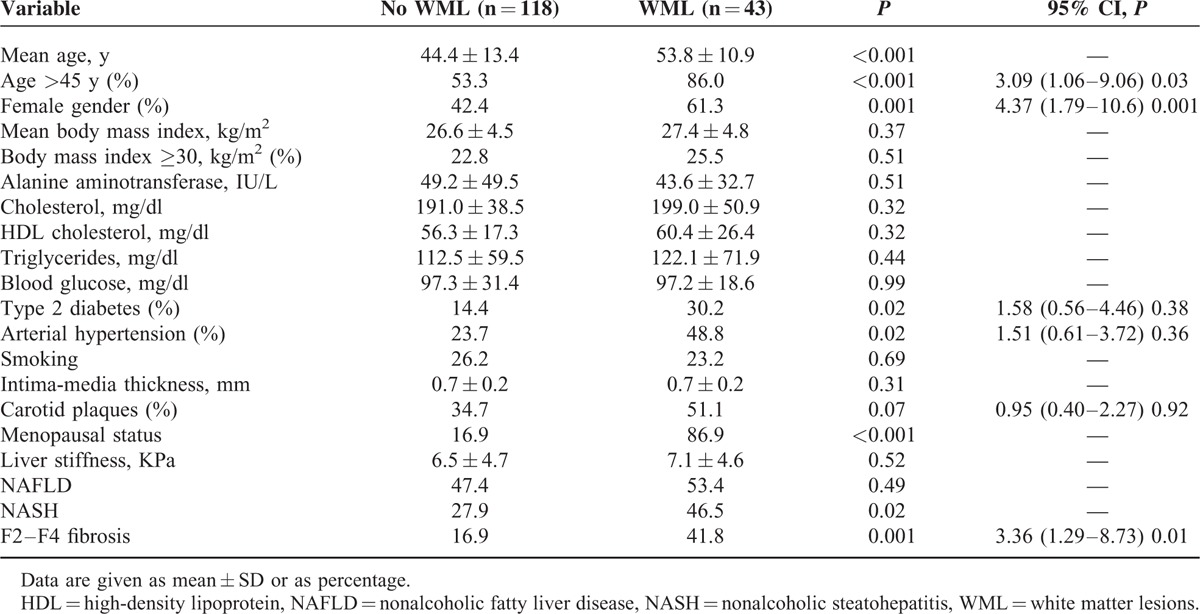
Univariate and Multivariate Analysis of Factors Associated With Presence of White Matter Lesions in the Entire Cohort of 161 Patients With and Without Nonalcoholic Fatty Liver Disease

Considering the higher risk of WML in females and in older subjects, we tested the association between menopausal status and WML in the female subset. Notably, we found that the prevalence of WML was higher in postmenopausal females when compared to fertile women (48.7% vs 28.5%, *P* = 0.06).

**FIGURE 2:**
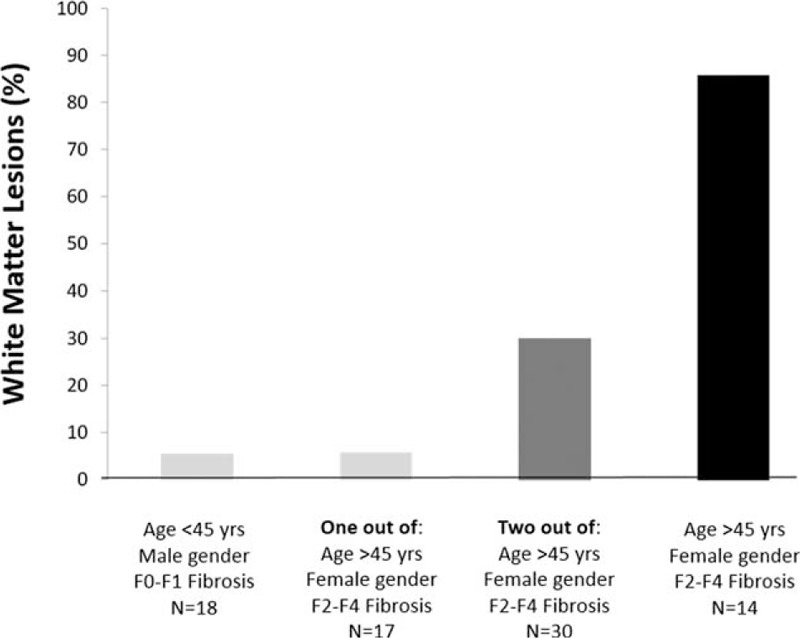
Prevalence of white matter lesions in patients with nonalcoholic fatty liver disease according to gender, age, and F2–F4 fibrosis.

In the subgroup of 79 patients with NAFLD we confirmed a higher prevalence of WML in patients with NASH (37.7% vs 11.5%; *P* = 0.01) and in those with F2–F4 fibrosis (47.3% vs 12.1%; *P* = 0.001) when compared with their counterpart. Again the presence of WML was associated with age ≥45 years (OR 11.1, 95% CI: 1.14–108.7; *P* = 0.03), female gender (OR 5.21, 95% CI: 1.39–19.6; *P* = 0.01) and presence of F2–F4 fibrosis (OR 5.34, 95% CI: 1.40–20.3; *P* = 0.01) (Table 3) by multivariate logistic regression analysis. When considering age as continuous instead of categorical variable we confirmed the independent link between F2–F4 fibrosis and WML (OR 5.26, 95% CI: 1.39–19.9; *P* = 0.01). When NASH was added in the model as independent variable, we confirmed F2–F4 fibrosis (OR 4.96, 95% CI: 1.08–22.7; *P* = 0.03), but not NASH (OR 1.18, 95% CI: 0.21–6.63; *P* = 0.84) as independent predictor of WML. Accordingly, the prevalence of WML progressively increased from patients without (1/18; 5.5%), or with 1 (1/17, 5.8%), to those with 2 (9/30; 30%) and further to those with 3 (12/14; 85.7%) risk factors (Figure 2).

**TABLE 3:**
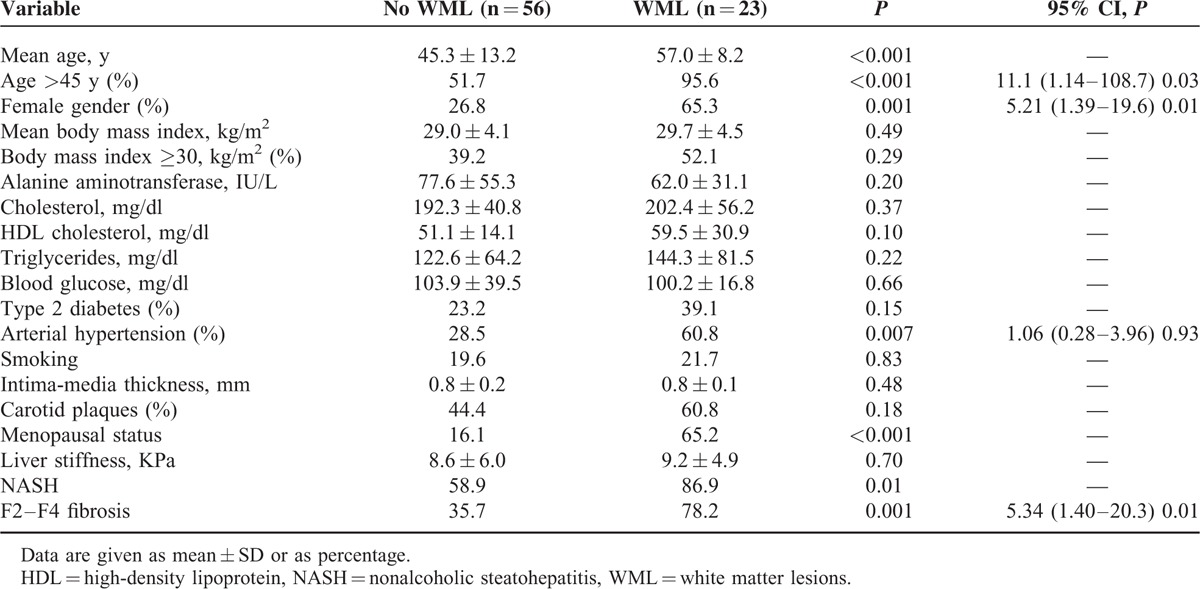
Univariate and Multivariate Analysis of Factors Associated With Presence of White Matter Lesions in 79 Patients With Nonalcoholic Fatty Liver Disease

As reported in the entire population, in the subset of female NAFLD the prevalence of WML was significantly higher in menopausal females when compared with fertile women (60.8% vs 14.2%, *P* = 0.03).

Finally, among control individuals, the presence of WML was independently linked to female gender (OR 4.46, 95% CI: 1.13–17.5; *P* = 0.03) and diabetes (OR 6.39, 95% CI: 1.14–35.6; *P* = 0.03).

### Mini-Mental State Examination and White Matter Lesions

MMSE was available in 128 patients (69 with and 59 without NAFLD), with similar characteristics compared to the entire cohort (data not shown). A pathological MMSE was observed in 12.5% (16/128) of patients, without significant difference between patients with (5/37; 13.5%) or without (11/91; 12%) WML (*P* = 0.82).

## DISCUSSION

In a mixed cohort of patients with NAFLD and of controls without fatty liver we observed that WML are not associated with the presence of NAFLD but with its fibrosis severity, also identifying in older age and female gender other relevant risk factors for WML. Of note, these associations were maintained after correction for cardiometabolic confounders, and kept in the separate analysis of NAFLD patients alone.

Different cross-sectional and prospective clinical studies reported an increase in subclinical cardiovascular dysfunctions^7–11^ and in cardiovascular events/mortality,^5,6,12,13^ including cerebrovascular ones, in patients with NAFLD compared with their counterpart. Along this line we looked at the relationship between WML—that is, subclinical vascular cerebral lesions—assessed by the gold standard MRI, and NAFLD. Notably we observed that WML were present in about 1 case in 4 in both the group of biopsy-proven NAFLD patients at high prevalence of NASH and significant fibrosis, and in control subjects without both fatty liver and liver damage. The observed prevalence of WML was within the wide range reported in literature.^18–23^ By contrast, when the entire cohort was stratified according to the presence of significant fibrosis, we found that the risk of having WML was about 2 times higher in patients with liver damage when compared with their counterpart. Notably, this issue was confirmed after adjusting for well-known cardiometabolic risk factors and in the subgroup of NAFLD patients. Thus, the severity of liver damage among NAFLD, not NAFLD per se, increases the risk of subclinical cerebrovascular lesions. Our data fully agree with growing literature showing higher cardiovascular damage in NAFLD according to the severity of liver fibrosis, and significant additive cardiovascular risk in NAFLD patients with liver damage only when compared to subjects without fatty liver. Specifically different studies reported an increase in carotid intima-media thickening,^10^ impaired kidney function,^33^ and impaired geometrical and functional cardiac indexes^34^ according to the severity of liver fibrosis in NAFLD. In addition, the severity of fibrosis in NAFLD, assessed by histology or by noninvasive scores, but not—or to a less extent—NAFLD per se, were reported as independent risk factors for the occurrence of diabetes^35^ and for cardiovascular mortality.^5,6,36^ In our study we did not find any significant association between NASH and WML. These data agree with 2 recent natural history study in NAFLD showing that only severity of fibrosis, not NASH predicted cardiovascular mortality in NAFLD.^5,6^

Notably, also in controls without fatty liver the importance of metabolic factors on predicting WML was strong; cerebral lesions were associated with the presence of diabetes, in addition to female gender.

Another relevant finding of our study lies in the independent association between WML and female gender among the entire cohort and in the subgroup of NAFLD patients. We are not able to fully explain mechanisms underlying this association, but we can suggest a relevant role of menopausal status in females. This condition is a well-recognized cardiovascular risk factor and recent studies also suggested an independent effect on the severity of liver damage in NAFLD.^37,38^ Therefore, the higher prevalence of WML we found in females, and the association, among females, between WML and menopausal status is not surprising. This result is in line with the increased stroke risk reported in women on menopausal status,^39^ and can account for the lack of the protective vascular effect exerted by estrogens.^40^

Our study is merely observational and not designed to explore the reasons for the association of WML with liver fibrosis. However, we may put forward a few hypotheses, leaving the demonstration of pathophysiological mechanisms to experimental studies. The reported association might stem from the proinflammatory, proatherogenic, and profibrogenic environment characterizing patients with NASH and significant fibrosis. This inflammatory state might be able to act systemically, affecting the homeostasis of different organs, including small cerebral vessels,^2^ as already demonstrated for systemic atherosclerosis, kidney, and heart damage.^10,33,34^ A direct relationship between an inflammatory background and WMLs is still lacking but some neuropathologic observations in infant leukoaraiosis^41^ further support the pathogenic link between inflammation, periventricular leukomalacia, and WML.

From a clinical point of view and provided our data receive external validation in independent cohorts, the present results suggest that among NAFLD the presence of significant liver damage, especially at older age and/or in females, identifies NAFLD patients at higher risk of WML. Notably we showed that the risk of having WML is negligible in absence of or with only one risk factor, significantly increasing in patients with two risk factors, and being very high in older females with significant liver fibrosis. The clinical meaning of these data and their implication prompts to a more intensive follow-up in some subgroups of patients. In our cohort WML were not associated with cognitive alterations evaluated by the MMSE, however the presence of WML might have clinical relevance and impact on the future development of stroke, cognitive decline, dementia, disability, and mortality.^16,17^ Notably, this is true also in individuals with mild grade WML,^42,43^ the most observed alteration in our cohort.

The study has both strengths and limits. The strength of our study lies in the availability of data on WML assessed by the gold standard MRI in a cohort of subjects with and without steatosis, even if WML were found in only 43 individuals. The main limitation is its cross-sectional nature, unable to prove the underlying pathogenic mechanisms(s) linking WML and liver fibrosis. A further methodological question is the potentially limited external validity of the results for different NAFLD populations and settings. Our study included a cohort of Italian NAFLD patients at high prevalence of NASH and severe fibrosis, who might be different, in terms of both metabolic features and of liver disease severity, from the majority of prevalent NAFLD cases in the general population. Another limitation is the use of ultrasonography, a less sensitive tool than liver biopsy to detect fatty infiltration, to exclude steatosis in the control group. The lack of matching for well-known cardiometabolic risk factors between NAFLD and controls without steatosis is a further limitation of our study. Adjusting by multivariate analysis however might attenuate this bias. Nevertheless a too strict matching could be responsible for a not fully evaluation of the role of comorbidities on WMLs pathogenesis. Another limit lies in the lack of a control group of patients with viral chronic hepatitis. In fact it should be possible that hepatic fibrosis per se, independently of the etiology of the liver disease, and not limited to NAFLD, can drive the risk for WML. Finally, we also need data on serum levels and on the hepatic expression of proinflammatory and profibrogenic cytokines potentially involved in the vascular alterations of NAFLD patients.

In conclusion, in a cohort from Southern-Italy, we showed that the presence of WML is not related with NAFLD per se but with its severity in terms of liver fibrosis. The mechanisms underlying these associations and their long-term clinical meaning need to be further investigated.
